# Integration of Data from NIPPON DATA80/90 and National Nutrition Survey in Japan: For Cohort Studies of Representative Japanese on Nutrition

**DOI:** 10.2188/jea.JE20090218

**Published:** 2010-05-05

**Authors:** Nagako Okuda, Katsuyuki Miura, Katsushi Yoshita, Yasuhiro Matsumura, Akira Okayama, Yasuyuki Nakamura, Tomonori Okamura, Shigeyuki Saitoh, Kiyomi Sakata, Toshiyuki Ojima, Tanvir Chowdhury Turin, Hirotsugu Ueshima

**Affiliations:** 1 Department of Health Science, Shiga University of Medical Science, Otsu, Japan; 2 Project for the National Health and Nutrition Survey, National Institute of Health and Nutrition, Tokyo, Japan; 3 Faculty of Healthcare, Kiryu University, Midori City, Japan; 4 The First Institute for Health Promotion and Health Care, Japan Anti-Tuberculosis Association, Tokyo, Japan; 5 Cardiovascular Epidemiology, Kyoto Women’s University, Kyoto, Japan; 6 Department of Preventive Cardiology, National Cardiovascular Center, Osaka, Japan; 7 Department of 2nd Internal Medicine, Sapporo Medical University, Sapporo, Japan; 8 Department of Hygiene and Preventive Medicine, Iwate Medical University, Morioka, Japan; 9 Department of Community Health and Preventive Medicine, Hamamatsu University School of Medicine, Hamamatsu, Japan

**Keywords:** nutrition, epidemiology, Japan

## Abstract

**Background:**

Diet is one of the most important lifestyle factors that a affect healthy life expectancy through onset of various lifestyle-related diseases. Large-scale cohort studies with sufficient baseline nutritional information are scarce. NIPPON DATA80/90 is cohort study of representative Japanese population, and the cohorts also participated in the National Nutrition Survey in Japan (NNSJ) at the baseline. The corresponding datasets could be combined.

**Methods:**

Individual records of NIPPON DATA and NNSJ were compared and integrated. Intakes of nutrients and food groups for individual participants were calculated by distributing intakes in the each household in NNSJ, considering age and sex of the individuals. The results from an international cooperative epidemiological study (INTERMAP) were utilized to estimate intakes of 75 nutrients for NNSJ80 and 70 nutrients for NNSJ90. Nutrient intakes calculated utilizing INTERMAP data were compared with those in the NNSJ datasets.

**Results:**

NIPPON DATA80/90 datasets were enhanced with detailed baseline nutrient intake data (the numbers of participants combined were 10 422 and 8342 for NIPPON DATA80 and 90, respectively). The mean nutrient intakes calculated through utilizing INTEMRAP data and those calculated from the NNSJ datasets were similar, and the calculated values were strongly correlated with those calculated from NNSJ datasets (Pearson’s correlation coefficients greater than 0.8 [*P* < 0.001]). Detailed nutrient intakes (eg, cholesterol, fatty acids, amino acids, and dietary fiber) were complemented.

**Conclusions:**

The nutrient intakes calculated from NNSJ datasets for the participants of NIPPON DATA are appropriate as the baseline nutrient intake data. The enhanced cohort datasets are suitable for investigations of baseline dietary habits and the consequent health status.

## INTRODUCTION

Diet is one of the most important factors that affects a healthy life expectancy through its association with the onset of cardiovascular diseases (CVD) and other lifestyle-related diseases.^1^^,^^2^ The National Integrated Project for Prospective Observation of Non-communicable Disease And its Trends in Aged (NIPPON DATA) is a set of cohort studies of representative Japanese; the baseline cohorts were the participants of the 3rd and 4th National Survey on Circulatory Disorders, Japan (NSCD), conducted in 1980 and 1990, respectively.^3^ The effects of CVD risk factors on mortality and impaired activities of daily living in Japan have been investigated through this project.^4^^,^^5^ Several dietary factors are known to be associated with CVD risk factors,^6^^,^^7^ and it is necessary to clarify the associations between dietary factors and CVD risk for the prevention of CVD by adopting favorable dietary habits.

The NIPPON DATA80 and 90 cohorts were also participants in the National Nutrition Survey in Japan (NNSJ) conducted in the same year as the NIPPON DATA baseline survey (the 3rd and 4th NSCD), in which weighing record method was employed and nutrient intakes were calculated for each household. In this study, we estimated nutrient intakes of each household member of NNSJ by proportional distribution method, and combined NIPPON DATA datasets with corresponding NNSJ datasets to produce integrated cohort datasets with baseline nutrient intake data.

Intakes of limited nutrients were calculated and published for NNSJ80 and 90^8^^,^^9^; however, more nutrients, ie, dietary cholesterol, n-3 polyunsaturated fatty acids, potassium, etc. are known to have effects on CVDs,^10^^,^^11^ so it is desirable to add them to the nutrient data. We supplemented nutrient intakes relevant to the CVD risk or CVD risk factors applying the INTERMAP Food Table in Japan, an integrated food database developed for an international cooperative epidemiological study.^12^^–^^15^ With these integrated cohort datasets including detailed dietary variables, dietary features promoting healthy life expectancy can be investigated.

In this paper, we describe the methods by which we estimated individual nutrient intakes for NNSJ datasets, combined NIPPON DATA and NNSJ datasets, and supplemented them with nutrient data necessary to investigate the dietary effects on CVD.

## METHOD

### NIPPON DATA80, 90

NIPPON DATA80/90 are follow-up studies of mortality from CVD and activities of daily living in representative Japanese men and women aged 30 years and older, and the complete details of the study population have been described elsewhere.^16^^,^^17^ Participants in NIPPON DATA80 and 90 were those persons surveyed for the 3rd and 4th NSCD, conducted in 1980 and 1990, respectively. They were the residents of 300 randomly selected districts throughout Japan. The surveys consisted of history-taking, physical examinations, blood tests, and a self-administered questionnaire on the lifestyle, including dietary habits using the food-frequency method. The numbers of follow-up participants were 10 546 for NIPPON DATA80, and 8383 for NIPPON DATA90. A follow-up survey was conducted first in 1994 for NIPPON DATA80, and in 1995 for NIPPON DATA90. Follow-ups have subsequently been conducted every 5 years, and the vital status, causes of death, and activities of daily living have been surveyed.

### National Nutrition Survey in Japan

National Nutrition Survey in Japan (NNSJ) has been conducted involving households in randomly selected survey areas throughout Japan every year since 1948, and the details of the survey have been described elsewhere.^18^^,^^19^ In the decennial NSCD years, NNSJ has been conducted employing the same participants as the NSCD. Three hundred areas throughout Japan were randomly selected in 1980 and 1990. All residents in the areas: 6400 households in 1980 and 6000 households in 1990, were the participants of the survey.

A dietary survey was carried out employing the weighing record method for three consecutive days in each household. Trained dietitians visited participants, and they were asked to weigh and record all foods and beverages that any of the household members consumed during the survey period. Dietitians visited participants’ homes at least once a day and confirmed the records during the survey.

Dietary records were coded using the Standard Tables for Foods in Japan, 3rd edition^8^ for NNSJ80 and 4th edition^9^ for NNSJ90, and intakes of nutrients and food groups were calculated for every household. The dietary records did not include information on the portion of each food that each household member actually ate. Nutrient intakes per person were calculated as intakes per household simply divided by the number of household members, and the values were used for analysis and tabulated for annual reports for NNSJ.^8^^,^^9^

Since NNSJ conducted in 1995, information on food consumption of individual households was collected and used to estimate nutrient intakes of individual household members.^20^^,^^21^ Averaged nutrient and food group intakes by sex and age group have been published since then.

We obtained NNSJ80 and NNSJ90 datasets from the Ministry of Health, Labour, and Welfare, which included nutrient and food group intakes per household, and the anthropometrics of individual household members. The NNSJ90 dataset included some nutrients that were not published in the annual report, eg vegetable protein, phosphorus, potassium, cholesterol, etc. (Table 1).

**Table 1.** Nutrient compositions calculated as representatives of food groups in the National Nutrition Survey in Japan using INTERMAP Food Table Japan and INTERMAP Japan recall data

	Unit	Publication in NNSJ80	Publication in NNSJ90	Source for integrated NIPPON DATA80	Source for integrated NIPPON DATA90
Total energy	kcal/100 g	Y^a^	Y	NNSJ^d^	NNSJ
Total protein	g/100 g	Y	Y	NNSJ	NNSJ
Animal protein	g/100 g	N^b^	Y	RCF^e^	RCF
Vegetable protein	g/100 g	N	C^c^	RCF	RCF
Amino acids (18 items)	mg/100 g	N	N	RCF	RCF
Total fat	g/100 g	Y	Y	NNSJ	NNSJ
Cholesterol	mg/100 g	N	C	RCF	RCF
Saturated fatty acid	g/100 g	N	C	RCF	RCF
Polyunsatudated fatty acid	g/100 g	N	C	RCF	RCF
Monounsaturated fatty acid	g/100 g	N	C	RCF	RCF
Fatty acids (42 items)	mg/100 g	N	N	RCF	RCF
Total carbohydrate	g/100 g	Y	Y	NNSJ	NNSJ
Starch	g/100 g	N	N	RCF	RCF
Sucrose	g/100 g	N	N	RCF	RCF
Avairable carbohydrate	g/100 g	N	N	RCF	RCF
Total dietary fiber	g/100 g	N	C	RCF	RCF
Phosphorus	mg/100 g	N	C	RCF	NNSJ
Calcium	mg/100 g	Y	Y	NNSJ	NNSJ
Iron	mg/100 g	Y	Y	NNSJ	NNSJ
Potassium	mg/100 g	N	C	RCF	NNSJ
Sodium	mg/100 g	Y	Y	NNSJ	NNSJ
Magnesium	mg/100 g	N	C	RCF	NNSJ
Vitamin A	IU/100 g	N	C	NNSJ	NNSJ
Vitamin B1	mg/100 g	Y	Y	NNSJ	NNSJ
Vitamin B2	mg/100 g	Y	Y	NNSJ	NNSJ
Vitamin C	mg/100 g	Y	Y	NNSJ	NNSJ
Vitamin E	mg/100 g	N	C	RCF	NNSJ
Niacin	mg/100 g	N	C	RCF	NNSJ

^a^Y: data published in NNSJ annual report.

^b^N: data not published in NNSJ annual report.

^c^C: calculated data included in NNSJ dataset but not publised in the annual report.

^d^NNSJ: National Nutrition Survey for Japan.

^e^RCF: Representative compositions for food groups.

### Estimation of nutrient intakes of individuals by calculating proportional distribution, and integration of NIPPON DATA and NNSJ datasets

**Figure 1.**
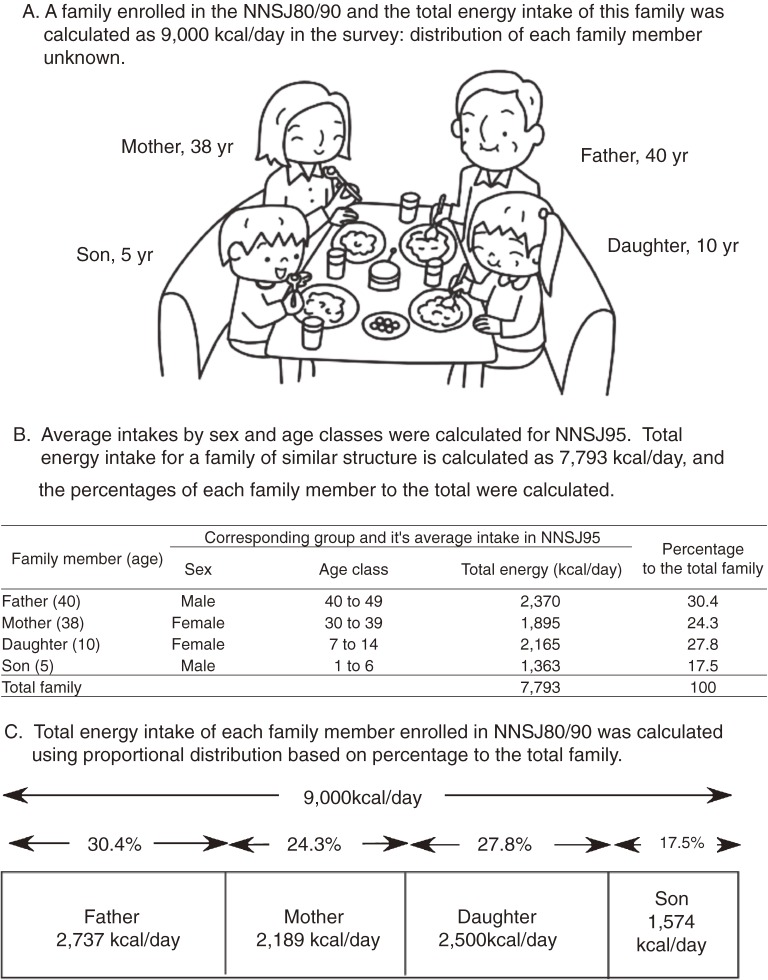
Calculation of nutrient intakes of individual household members based on the total intake of the household by proportional distribution (an example using total energy)

We estimated the nutrient intakes of each household member by dividing the household intake data of NNS80 and 90 using average intakes by sex and age groups calculated for NNSJ95. The average intakes in NNSJ95 were calculated by a combined method using household-based food-weighing records and an approximation of the proportions of each dish or food shared in the household.^21^ An example of proportional distribution calculation for total energy intake is summarized in Figure 1. Other nutrient and food group intakes of individuals were calculated in the same manner.

For nutrients used as energy sources, densities were calculated as %kcal in both proportionally distributed and simple averaged densities. For other nutrient and food group intakes, densities were calculated as g/1000 kcal, mg/1000 kcal, etc. Simple averaged densities of intakes for nutrients and food groups were also estimated by dividing the intakes of the household by the total energy intake.

Individual records in corresponding NIPPON DATA and NNSJ datasets were compared based on the area code, household code, age, sex, height, and weight. Individuals in NIPPON DATA and NNSJ were combined if they were identified as the same person.

### Enhancement of nutrients using INTERMAP food database

Intakes of energy, macronutrients, and other nutrients (calcium, sodium, iron, and vitamins A, B1, B2, and C) were calculated and published in the annual reports of NNSJ80 and 90.^8^^,^^9^^,^^18^ We included other nutrients known to have effects on CVD risk factors by utilizing food group intakes data per household in NNSJ80 and 90 datasets, the INTERMAP Food Table, and INTERMAP Japan dietary survey data. These nutrients are shown as those calculated using RCF (representative compositions for food groups, described below) in Table 1.

INTERMAP (the International Study of Macro- and Micro-nutrients and Blood Pressure) is an international cooperative epidemiological study, and the participants were men and women ages 40 to 59 from 17 centers in four countries (Japan, China, UK, and US). Details of the study have been described elsewhere.^12^^,^^14^ Dietary data were obtained through four times of 24-hr dietary recalls conducted for each participant with internationally standardized quality control procedures. We used dietary recall data for participants of the INTERMAP Japan survey to calculate representative food consumption patterns in Japan.

The INTERMAP Japan survey was conducted from 1996 to 1998, and the participants were 1145 men and women ages 40 to 59, randomly selected in four research centers in Japan (299 from Sapporo, 297 from Toyama, 258 from Aito, and 288 from Wakayama). A total of 277 638 food intakes were recorded for the 4580 dietary recalls, and these foods were coded using the INTERMAP Food Table, an integrated food database based on Standard Tables for Japanese Food, 4th edition.^13^^,^^22^ The INTERMAP Food Table consists of 2931 foods, and all foods are enhanced with a detailed nutrient composition not fully tabulated in the Standard Tables for Japanese Food, 4th edition, ie, cholesterol, fatty acids (42 items including trans fatty acids), amino acids (18 items), dietary fiber, magnesium, zinc, copper, selenium, retinol, sucrose, starch, and available carbohydrate.^13^

We calculated a representative nutrient composition for food groups (RCF) used in NNSJ using INTERMAP recall data as an average food consumption pattern of the Japanese population. Namely, foods recorded in the INTERMAP dietary recalls were assigned to any of the 89 (NNSJ80) or 85 (NNSJ90) food groups, proportions of foods making up each food group were calculated, and RCF for NNSJ80 and 90 were calculated as the weighted mean of constituent foods.

Nutrient intakes for households were calculated using food group intakes in NNSJ and RCF. Nutrient intakes for individual household members were calculated by proportional distribution using average intakes by age and sex class in NNSJ95, as mentioned above (Figure 1).^20^

Means of nutrient intakes of individuals calculated by the proportional distribution from the original NNSJ dataset and re-calculation using RCF were compared using *t*-tests. Correlations between nutrient intakes of individuals calculated from NNSJ datasets and those estimated using RCF were examined using Pearson’s correlations coefficient. The significance level was set at 0.05. All analyses were performed using SPSS for Windows 15.00 (SPSS Inc., Chicago, IL, USA).

## RESULTS

NNSJ80 included nutrition survey data from 6456 households, and NNSJ90 included data from 5561 households. Nutrient intakes of individual family members based on the proportional distribution were calculated for 22 341 and 17 986 individuals for NNSJ80 and 90, respectively. Simple densities were also calculated by dividing nutrient intakes per household by 1000 kcal. The numbers of individuals aged 30 years and over were 12 947 and 11 196 for NNSJ80 and NNSJ90, respectively. Individual records in corresponding NIPPON DATA and NNSJ datasets were compared. A total of 10 422 individuals in NNSJ80 and 8342 individuals in NNSJ90 were identified as the same person, and their data were combined.

RCF for NNSJ80 and 90 with detailed nutrients was calculated using food tables and dietary recall data for the INTERMAP Japan Study. The nutrients included in the RCF are listed in Table 1. The mean nutrient intakes of individuals calculated for NIPPON DATA80/90 are tabulated in Appendix tables. The mean major nutrient intakes of individuals calculated by proportional distribution from the original NNSJ80 data (a) and estimated values using RCF80 (b) were compared for NNSJ80 (Table 2). Percent differences of (b) from (a) were within ±5% for total energy and macronutrients. The percent difference was over ±10% only for the iron intake. Correlation coefficients for the nutrient intakes of individuals between those calculated from NNSJ80 and from RCF are shown in Table 3. The values were highly correlated for all nutrients; all coefficients were greater than 0.9 (*P* < 0.0001).

**Table 2.** Comparison of nutrient intakes of individuals calculated by proportional distribution from (a) NNSJ80 dataset and (b) calculated values using representative compositions for food groups (RCF). Follow-up participants for NIPPON DATA80, 10 422 men and women, aged 30 years and over, 1980, Japan

	NNSJ80		RCF80		% difference of (b) from (a)
	
	Mean (a)	(SD)	Mean (b)	(SD)	
Total energy (kcal/day)	2137	(506)	2156	(510)	+0.9
Total protein (%kcal)	15.3	(2.1)	16.0	(2.1)	+4.5
Total fat (%kcal)	21.0	(5.6)	21.7	(5.1)	+3.5
Carbohydrate (%kcal)	61.0	(6.8)	58.7	(6.6)	−3.8
Sodium (mg/1000 kcal)	2588	(851)	2439	(773)	−5.8
Calcium (mg/1000 kcal)	255	(70)	258	(69)	+1.0
Iron (mg/1000 kcal)	6.7	(1.3)	5.4	(0.9)	−19.4

NNSJ, National Nutrition Survey in Japan; RCF, Representative compositions for food groups.

Nutrient intakes of each family member were estimated by dividing the household intake data of NNS80 using average intakes by age and sex groups calculated for NNSJ95.

**Table 3.** Correlations between nutrient intakes of individuals estimated from (a) original NNSJ80 and (b) calculated values using representative nutrient composition for food groups. Follow-up participants for NIPPON DATA80, 10 422 men and women, aged 30 years and over, 1980, Japan

	*r*^a^	*P*
Total energy (kcal/day)	0.986	<0.001
Total protein (%kcal)	0.922	<0.001
Total fat (%kcal)	0.944	<0.001
Carbohydrate (%kcal)	0.951	<0.001
Sodium (mg/1000 kcal)	0.969	<0.001
Calcium (mg/1000 kcal)	0.955	<0.001
Iron (mg/1000 kcal)	0.906	<0.001

^a^Pearson’s correlation coefficient.

NNSJ: National Nutrition Survey in Japan.

Nutrient intakes of each family member were estimated by dividing the household intake data of NNSJ80 using average intakes by age and sex groups calculated for NNSJ95.

For NNSJ90, the average intakes of individuals calculated from the original NNSJ90 data (a) and estimated values using RCF90 (b) were compared (Table 4). Percentage differences of (b) from (a) were within ±5% for total energy, total fat, carbohydrates, iron, potassium, and the P/S ratio; within ±10% for total protein, calcium, vegetable protein, cholesterol, saturated fatty acids, monounsaturated fatty acids, and polyunsaturated fatty acids. The average animal protein intake calculated using RCF90 was 18% higher than the value using NNSJ90. Table 5 shows the correlation coefficients for nutrient intakes of individuals between those calculated from NNSJ90 and RCF90. Correlation coefficients were greater than 0.9 (*P* < 0.0001) for total energy, total protein, total fat, carbohydrates, potassium, animal protein, cholesterol, monounsaturated fatty acids, and polyunsaturated fatty acids, and all coefficients were greater than 0.8 (*P* < 0.0001).

**Table 4.** Comparison of nutrient intakes of individuals calculated by proportional distribution from (a) NNSJ90 dataset and (b) calculated values using representative nutrient compositioon for food groups. Follow-up pariticipants for NIPPON DATA90, 8342 men and women, aged 30 years and over, 1990, Japan

	NNSJ90		RCF90		% difference of (b) from (a)
	
	Mean (a)	(SD)	Mean (b)	(SD)	
Total energy (kcal/day)	2050	(467)	2026	(458)	−1.2
Total protein (%kcal)	15.8	(2.0)	16.8	(2.1)	+6.2
Total fat (%kcal)	23.5	(4.9)	23.2	(4.6)	−1.3
Carbohydrate (%kcal)	58.0	(6.1)	56.5	(5.9)	−2.6
Calcium (mg/1000 kcal)	266.7	(83.2)	286.3	(79.5)	+7.3
Iron (mg/1000 kcal)	5.8	(1.2)	5.8	(1.0)	−1.4
Sodium (mg/1000 kcal)	2633	(750)	2439	(648)	−7.3
Potassium (mg/1000 kcal)	1415	(299)	1435	(274)	+1.4
Animal protein (%kcal)	8.1	(2.0)	9.5	(2.0)	+18.0
Vegetable protein (%kcal)	7.7	(1.0)	7.2	(1.0)	−7.1
Cholesterol (mg/1000 kcal)	183	(57.39)	193	(57.39)	+5.7
Saturated fatty acids (%kcal)	6.75	(1.71)	6.24	(1.50)	−7.6
Monounsaturated fatty acids (%kcal)	7.72	(1.89)	8.34	(1.91)	+8.0
Polyunsaturated fatty acids (%kcal)	6.32	(1.42)	5.86	(1.49)	−7.3
P/S ratio	0.970	(0.224)	0.975	(0.260)	+0.6

NNSJ, National Nutrition Survey in Japan; RCF, Representative compositions for food groups; P/S, polyunsaturated fatty acids/saturated fatty acids.

Nutrient intakes of each family member were estimated by dividing the household intake data of NNS90 using average intakes by age and sex groups calculated for NNSJ95.

**Table 5.** Correlations between nutrient intakes of individuals estimated from (a) NNSJ90 and (b) calculated value using representative nutrient compositions for food groups. Follow-up pariticipants for NIPPON DATA90, 8342 men and women, aged 30 years and over, 1990, Japan

	*r*^a^	*P*
Total energy (kcal/day)	0.983	<0.001
Total protein (%kcal)	0.916	<0.001
Total fat (%kcal)	0.933	<0.001
Carbohydrate (%kcal)	0.956	<0.001
Calcium (mg/1000 kcal)	0.874	<0.001
Iron (mg/1000 kcal)	0.807	<0.001
Sodium (mg/1000 kcal)	0.821	<0.001
Potassium (mg/1000 kcal)	0.917	<0.001
Animal protein (%kcal)	0.936	<0.001
Vegetable protein (%kcal)	0.868	<0.001
Cholesterol (mg/1000 kcal)	0.924	<0.001
Saturated fatty acids (%kcal)	0.892	<0.001
Monounsaturated fatty acids (%kcal)	0.915	<0.001
Polyunsaturated fatty acids (%kcal)	0.905	<0.001
P/S ratio	0.831	<0.001

^a^Pearson’s correlation coefficient.

NNSJ, National Nutrition Survey in Japan; P/S, polyunsaturated fatty acids/saturated fatty acids.

Nutrient intakes of each family member were estimated by dividing the household intake data of NNS90 using average intakes by age and sex groups calculated for NNSJ95.

Medians were also calculated for NNSJ80 and 90, and the median values were somewhat lower (within five percent) than the mean values in most nutrients, showing slightly skewed, nonnormal distributions with right tails (data not shown). Some of the estimated nutrient intakes were extremely higher or lower as intakes of a general adult, ie, total energy intakes were less than 500 kcal/day for seven participants and one participants in NNSJ80 and 90, respectively, and more than 5000 kcal/day for eight participants and one participant in NNSJ80 and 90, respectively (data not shown).

## DISCUSSION

In this study, NIPPON DATA80 and 90 were enhanced with baseline nutrient intake data of individuals by combining the nutrient intakes of individuals calculated from the corresponding NNSJ dataset. The nutrient intakes of individual household members were estimated by calculating the proportional distributions of the intakes per household concerning the age and sex of the individual members. To our knowledge, there is no study that used this method for researches in nutritional epidemiology. However, a similar method was applied to estimate disease-specific costs using health insurance claims in a health economic study showing good valitidy.^23^^,^^24^ In this study, The values were calculated as weighted averages using the average nutrient intake pattern according to the sex and age in 1995, and individual habits in the food distribution within the household were not considered, ie, a household in which the husband ate fish and wife did not. However, familial resemblance in nutrient intake has been reported in spouses, and also in parents and their children,^25^^–^^27^ and we think that the proportional distribution method used in this study is appropriate for most households. Distributions of the estimated nutrient intakes were checked, and the median values were slightly lower than the mean values. This kind of nonnormal distribution is often observed in many nutrients, and the distribution pattern is also different by nutrients.^28^ In our further researches, the nonnormal distribution of nutrient intake should be taken into consideration. Some estimated nutrient intakes were extremely higher or lower as intakes of a general adult, and these outliers are kept in the combined datasets. Cutoff values should also be considered according to the objective of analysis.

Studies on nutrient intakes of Japanese with average intakes by age classes in the 1980s are scarce. There is a possibility that the food distribution pattern among generations might have changed from 1980 to 1995. In Japan, the major trend in postwar dietary habits has been westernization, ie, an increase in intakes of meat, milk and dairy products, and a decrease of rice, and fish, bringing about an increase of serum cholesterol level in Japanese.^29^^,^^30^ Generally, younger people are more sensitive to change, and westernization of the diet would have had a more prominent effect on the younger generation than those born earlier, which is concordant with the trend in the serum cholesterol level of Japanese in the postwar period; a higher cholesterol level in those born later.^31^^,^^32^ In NNSJ, a more westernized diet in younger people (more meat and less fish than the elderly) was observed in both 1995^20^ and 2005.^29^ It might well be that dietary westernization has occurred in the younger generation first, influencing the older generation, and those people getting older, since the postwar era to the present in Japan. It can be inferred that the basic order in the food distribution among generations remained mostly unchanged in this period, and the proportional distribution using average intakes in NNSJ95 is fairly reasonable.

Stroke mortality in Japan was highest in 1960s, and it has declined drastically since then without rising coronary heart disease mortality, with Japanese diet drawing attention as a healthy diet.^33^^–^^35^ Some features of the Japanese diet have already been suggested to influence CVD risks, ie, a higher salt intake associated with a higher blood pressure,^36^ and a higher long chain n-3 polyunsaturated fatty acid intake associated with a lower incidence of coronary heart disease.^33^ The original NNSJ datasets were not thought to be sufficient for investigation of the nutrition-disease relation, because of the limited kinds of nutrients, and should be enhanced with other nutrients. The INTERMAP Food Table Japan and INTERAMP Japan recall data were used to calculate RCF for NNSJ, and to enhance nutrients of the NNSJ. The INTERMAP Food Table was enhanced with detailed data on nutrient compositions, ie, fatty acids and amino acids.^13^ The RCF included these detailed nutrients, and the intakes were calculated for the participants.

In the present report, the nutrient intakes of individuals calculated from the NNSJ dataset and RCF were compared. The mean iron intake calculated from NNSJ80 was 6.7 mg/day, and the value calculated from RCF was 5.4 mg/day, 20% lower than former (Table 2). However, the correlation coefficient between intakes by the two methods was large (*r* = 0.919, Table 3). The lower iron intake estimated from RCF seemed to be related to the food table used to calculate nutrient intakes for NNSJ. For NNSJ80, the 3rd revised edition of the standard tables of food composition in Japan was used, and the 4th edition was used for the NNSJ conducted in 1985. Comparisons of nutrient intakes using NNSJ82 data were made, in which nutrient intakes were calculated using the 3rd and 4th editions of the food table.^37^ In this report, the total energy intake calculated using the 4th edition was 1.7% higher than the value calculated with the 3rd edition, 5.6% higher for total fat intake, and 19.4% lower for iron intake. The difference in iron intake is consistent with our results.

For other nutrients, mean intakes calculated with RCF using INTERMAP data showed some differences compared with the values obtained from NNSJ (Tables 2 and 4). Possible reasons for these differences are as follows. First, the INTERMAP Japan survey was conducted in 1996–98, years after NIPPON DATA80 and 90. The food consumption pattern constituting the food groups might have changed due to alterations of the dietary habit during this period in Japan.^38^ Second, INTERMAP participants were aged 40–59, and those of NNSJ were all residents in the surveyed area. Children, young people, and the elderly were not included in INTERMAP. The dietary characteristics of these people were therefore not reflected in the RCF.

These factors might have caused the differences between nutrient intakes calculated by RCF and those obtained from the original NNSJ. However, the nutrient values of individuals calculated with RCF were highly correlated with those values calculated from NNSJ (Tables 3 and 5), which may indicate that RCF was appropriate for both animal and vegetable foods, and the estimated detailed nutrient intakes of individuals are useful for the investigation of dietary factors affecting the CVD risk within the cohort. However, some attention is necessary when we assess changes in nutrient intakes over time.

Although alcohol intake is an important dietary factor,^39^^,^^40^ it was not included in the integrated datasets. Intakes of alcoholic beverages were recorded for NNSJ, and the alcohol intake per household was included in the NNSJ datasets. However, we thought that calculation of the alcohol intake of individuals from the intake per household would not be appropriate for investigation of the alcohol-disease association, due to the large inter-individual differences in alcohol consumption within a household. Information on alcohol consumption could not be enhanced in this study. It would be appropriate to use data derived from a question asking about the frequency of alcohol consumption in NIPPON DATA.

The combination of nutrient values obtained from NNSJ datasets and those calculated with RCF based on the INTERMAP Japan survey would be appropriate for the investigation of dietary factors affecting CVD risk. The adopted sources of detailed nutrient intakes are tabulated in Table 1, decided on considering the consistency of nutrients with a more detailed classification; ie, saturated, monounsaturated, or polyunsaturated fatty acids and each fatty acid.

In conclusion, NIPPON DATA80/90, large-scale cohort datasets in Japan, were enhanced with the detailed baseline nutrient intake data obtained by the weighing record method in NNSJ80 and 90. Nutrients related to CVD risk factors, eg, potassium, cholesterol, n-3 polyunsaturated fatty acids, were also incorporated to enhance the datasets. The integrated cohort datasets would be useful for studies on CVD prevention through favorable dietary habits.
