# J Epidemiol 2010;20(Suppl 3):S506–S514 Integration of Data from NIPPON DATA80/90 and National Nutrition Survey in Japan: For Cohort Studies of Representative Japanese on Nutrition

**DOI:** 10.2188/jea.JE20090218E

**Published:** 2010-07-05

**Authors:** Nagako Okuda, Katsuyuki Miura, Katsushi Yoshita, Yasuhiro Matsumura, Akira Okayama, Yasuyuki Nakamura, Tomonori Okamura, Shigeyuki Saitoh, Kiyomi Sakata, Toshiyuki Ojima, Tanvir Chowdhury Turin, Hirotsugu Ueshima

In the Acknowledgments, there are two errors in the first names of members of the NIPPON DATA80/90 Research Group:

**Hiroshi** Kiyohara should be **Yutaka** Kiyohara; **Atsuhi** Hozawa should be **Atsushi** Hozawa.

The authors regret these errors.

